# Benign and Malignant Findings on Chest CT Among Adult Survivors of Childhood and Young Adult Cancer with a History of Chest Radiotherapy

**DOI:** 10.21203/rs.3.rs-2599972/v1

**Published:** 2023-02-21

**Authors:** Dana Barnea, Emily S. Tonorezos, Amber Khan, Joanne F. Chou, Chaya S. Moskowitz, Rana Kaplan, Kevin C. Oeffinger

**Affiliations:** Tel Aviv Sourasky Medical Center; National Cancer Institute; Montefiore Medical Center-Albert Einstein College of Medicine; Memorial Sloan Kettering Cancer Center; Memorial Sloan Kettering Cancer Center; Memorial Sloan Kettering Cancer Center; Duke University

**Keywords:** chest CT, cancer survivor, pulmonary nodule, lung cancer

## Abstract

**Purpose:**

Childhood and young adult cancer survivors exposed to chest radiotherapy are at increased risk of lung cancer. In other high-risk populations, lung cancer screening has been recommended. Data is lacking on prevalence of benign and malignant imaging abnormalities in this population.

**Methods:**

We conducted a retrospective review of imaging abnormalities in chest CTs performed more than 5 years post-cancer diagnosis in survivors of childhood, adolescent, and young adult cancer. We included survivors exposed to radiotherapy involving the lung field and followed at a high-risk survivorship clinic between November 2005 and May 2016. Treatment exposures and clinical outcomes were abstracted from medical records. Risk factors for chest CT-detected pulmonary nodule were assessed.

**Results:**

Five hundred and ninety survivors were included in this analysis; median age at diagnosis, 17.1 years (range, 0.4–39.8) and median time since diagnosis, 21.1 years (range, 0.4–58.6). At least one chest CT more than 5 years post-diagnosis was performed in 338 survivors (57%). Among these, 193 (57.1%) survivors had at least one pulmonary nodule detected on a total of 1057 chest CTs, resulting in 305 CTs with 448 unique nodules. Follow-up was available for 435 of these nodules; 19 (4.3%) were malignant. Risk factors for first pulmonary nodule were older age at time of CT, CT performed more recently and splenectomy.

**Conclusions:**

Benign pulmonary nodules are very common among long-term survivors of childhood and young adult cancer.

**Implications for Cancer Survivors:**

High prevalence of benign pulmonary nodules in cancer survivors exposed to radiotherapy could inform future guidelines on lung cancer screening in this population.

## Introduction

Lung cancer is the leading cause of death from cancer worldwide [1]. In recent years, this has resulted in ongoing efforts to reduce mortality by screening high-risk individuals using low-dose computed-tomography (LDCT). The National Lung Screening Trial was the first large trial to show a reduction in all-cause and lung cancer-specific mortality from LDCT screening [2]. Based upon the evidence from this study and others, in 2013, the United States Preventive Service Task Force (USPSTF) recommended (Grade B) LDCT for individuals aged 55–80 with a history of more than 30 pack-years (PY) of smoking [3]. Since then, several studies have reported on the effectiveness of this screening recommendation with demonstration of benefit and substantial false positive rates [4][5][6][7][8][9].

Cancer survivors exposed to radiotherapy including the lung field have an increased risk of lung cancer [10][11][12]. Radiotherapy is an integral part of the treatment regimen for many pediatric and young adult cancers, including Hodgkin lymphoma, non-Hodgkin lymphoma, Wilms tumor, sarcoma and with the use of total body irradiation as a preconditioning regimen for hematopoietic cell transplantations. In a cohort of long-term Hodgkin lymphoma survivors, the standardized incidence ratio (SIR) of lung cancer in survivors exposed to supra-diaphragmatic radiotherapy was 7.7 compared with the general population [11]. Smoking had a multiplicative interaction with radiotherapy; the hazard ratio of lung cancer among smokers who were exposed to radiotherapy was 14 compared with nonsmokers with no radiotherapy. In contrast to many other secondary malignancies, lung cancer risk begins to rise as early as one-year post-treatment [10]. Factors further increasing risks include older age at treatment and alkylating agents [10] [13][12]. Despite these known risks, whether screening for lung cancer in survivors of childhood and young adult cancer is appropriate and whether the harms of screening outweigh the benefits is unclear.

A crucial step in evaluating the utility of a screening recommendation is assessing the false positive rate. With regards to lung cancer screening, this would be affected by the frequency of imaging abnormalities detected by chest CT (computed tomography), ultimately deemed benign. To the best of our knowledge, there are no studies describing chest imaging abnormalities after chest radiotherapy for childhood or young adult cancer. In the general population not exposed to radiation, these imaging abnormalities are not rare. Gould et al described the incidence of pulmonary nodules in the setting of chest CTs performed for purposes other than screening and found that 29% of scans had at least one nodule [14]. Data describing such abnormalities in cancer survivors are lacking. Such information could be used to inform the clinical significance of imaging abnormalities and the possible harms associated with lung cancer screening in this unique population.

The purpose of this study was to evaluate the prevalence of imaging abnormalities on chest CTs performed in adult survivors of childhood and young adult cancer exposed to chest radiation followed in a high-risk survivorship clinic in our institution. Since many survivors undergo frequent chest imaging studies to detect recurrence or to evaluate respiratory symptoms, diagnostic CT scanning is common in this population. We describe imaging abnormalities found on these CT scans and their clinical outcome.

## Methods

The Adult Long-Term Follow-Up (LTFU) clinic at Memorial Sloan Kettering Cancer Center (MSKCC) in New York provides longitudinal risk-based health care for adult survivors of childhood and young adult cancer. Survivors are followed with a comprehensive visit once every 6–12 months. To be followed in the clinic, survivors must be over 18 and done with treatment for their primary cancer.

After Institutional Review Board approval, a retrospective chart review was conducted on all patients seen in the Adult LTFU clinic between November 1, 2005 and May 31, 2016 (n=1277). Inclusion criteria included survivors age 40 or younger at the time of diagnosis who received radiotherapy that included the lungs in the radiation field (i.e. mantle, hemi-thorax, whole lung, lung, mediastinum, axilla, chest, chest wall, thoracic vertebrae and total body irradiation). Exclusion criteria included patients with fewer than 5 years of follow-up since diagnosis. All data were obtained from review of the MSKCC medical record.

Demographics, therapeutic exposures, and clinical outcomes were abstracted. All chest CT scan reports performed more than 5 years post-diagnosis were reviewed, including studies performed outside of MSKCC when available. Two physicians (DB, AK) reviewed all reports and abstracted pulmonary abnormalities (e.g., pulmonary nodules, opacities and fibrosis). Each nodule received an identifying number and was followed through the consecutive CTs.

Descriptive statistics were used for demographics, treatment variables and clinical outcomes. Chest CTs were described beginning 5 years after diagnosis. Prevalence of chest CT findings were estimated among patients who have had at least one chest CT.

Risk factors associated with presence of first new pulmonary nodule were identified using a generalized estimating equation model assuming a Poisson distribution with a log-link function and an independent correlation matrix structure. A total of 827 records of chest CTs among 338 patients with at least one chest CT were included. The final model was constructed by including all univariable risk factors with p-value<0.2. Prevalence ratio (PR) and 95% confidence interval (CI) are presented.

All statistical analyses were performed using SAS version 9.3 (SAS Institute, Inc., Cary, NC, USA). All *P*-values were two-sided. *P*-values of <0.05 were considered to indicate statistical significance.

## Results

Five-hundred and ninety adult survivors had a history of radiotherapy including a lung field. Demographics, treatment exposures and smoking status are outlined in Table 1. Median age at primary cancer diagnosis was 17.1 years (range, 0.4–39.8), with a median follow-up of 21.1 years since diagnosis (range, 0.4–58.6). The most common cancer diagnosis was Hodgkin lymphoma. Approximately 16% of the cohort had ever smoked (n=92); six patients (1%) reported smoking more than 30 pack-years. A third of the survivors had undergone a hematopoietic stem cell transplant (n=186) and 14.8% (n=87) had undergone a splenectomy.

Of the 590 survivors, 338 (57%) had at least one chest CT scan performed more than 5 years following their primary cancer diagnosis. Over a median follow-up of 18.4 years, 1,444 chest CTs were performed. The median number of chest CTs among those who had at least one performed was 3 (range, 1–35).

**Figure 1:**
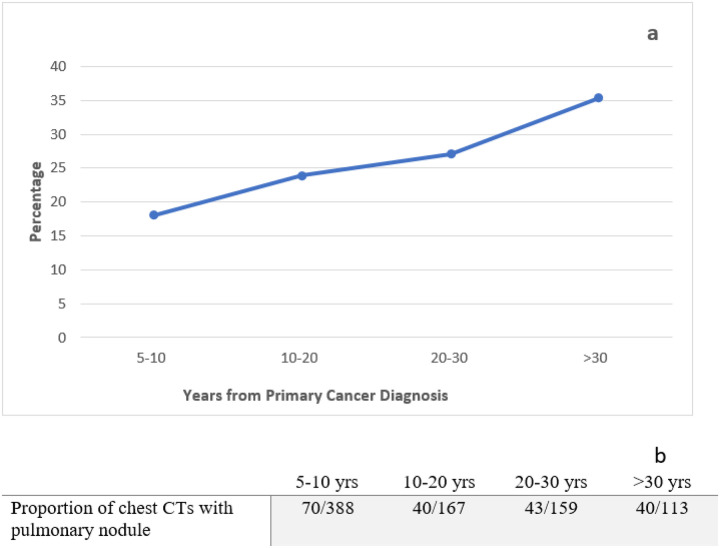
Proportion of first pulmonary nodule on chest CT by time since primary cancer diagnosis in adult survivors of childhood and young adult cancer exposed to chest radiation.

The prevalence of pulmonary abnormalities detected on chest CTs is shown in Table 2; 271 (80.1%) had any pulmonary abnormality noted and 193 (57.1%) had at least one lung nodule detected on their chest CT scan. Among those with at least one pulmonary nodule detected on chest CT, the median number of nodules was 2 (range, 1–13). The prevalence of a first pulmonary nodule increased as time from diagnosis passed. (Figure 1). Between 5–10 years post-diagnosis prevalence was approximately 18%, whereas after 30 years post-diagnosis prevalence was over 35%.

Clinical outcomes are described in Table 3. Of the 193 survivors with a pulmonary nodule, lung biopsy was performed in 18 survivors. Two patients had more than one biopsy. The remainder of the survivors with a CT-detected pulmonary nodule (n=175) were followed clinically and had no subsequent evidence of malignancy.

A malignancy was detected in 40% (8/20) of lung biopsies and an additional 5 malignancies were diagnosed via biopsy of a non-lung site and imaging suggestive of lung metastasis. Four of these 13 malignancies were pulmonary adenocarcinoma. Pulmonary metastases were diagnosed in the remaining 9 survivors: two represented recurrences of primary cancer diagnosis and 7 represented metastases of subsequent malignant neoplasms. Ultimately, two survivors died of lung cancer and six died of other cancers.

Among the 193 patients with at least one pulmonary nodule, 448 unique nodules were detected. Of these, outcome was available for 435 nodules: via biopsy in 20 nodules and via one-year follow-up data in 415 nodules. Only 19 (4.3%) of these 435 nodules were malignant.

Factors that were significantly associated with the presence of a first pulmonary nodule on chest CT were older age at time of CT, CT performed more recently and splenectomy. The data also suggest that there may be an association between smoking status (less than 30 PY vs 30 or more PY vs never smoker) and the prevalence of a nodule (PR=1.34 [95% CI: 0.96–1.87], p=0.056) although this was not statistically significant. In the final multivariable model, the PRs were 0.35 [95%CI: 0.04–2.92], 0.51 [95%CI: 0.31–0.82], 0.69 [95%CI: 0.51–0.94] for detection of a nodule on chest CTs performed between 1980–1990, 1991–2000 and 2001–2010, respectively, as compared with chest CTs performed after 2010. Survivors who had a history of a splenectomy were 57% more likely to have a pulmonary nodule detected (PR=1.57 [95%CI: 1.09–2.25], p=0.016) than survivors without a history of splenectomy. (Table 4)

## Discussion

This is the first report of chest CT findings and subsequent clinical outcomes in survivors of childhood and young adult cancer with a history of chest radiotherapy. Of 590 survivors exposed to chest radiation, 338 (57%) had at least one chest CT performed more than 5 years post diagnosis; of these, the majority 271 (80.1%) had a pulmonary abnormality noted and 193 (57.1%) had at least one pulmonary nodule. Most nodules detected were benign. During our follow-up, only 13 survivors (6.7 % of survivors with a CT-detected nodule) were diagnosed with malignancy in the lung.

The prevalence of pulmonary nodules on chest CTs performed in the general population has been estimated between 13.9%−31% [15][16][14]. These studies estimated nodule prevalence in chest CTs performed for reasons other than lung cancer screening. In these studies, the population included was older than our cohort. In one of the larger studies summarizing data from 200,000 members of the Kaiser Permanente South California Healthcare System, the prevalence of nodules was 29%, with a mean age of 63.4 years [14]. In that study nearly half of the patients with nodules were either current or former smokers, compared to 17.5% in our cohort. These findings suggest that childhood and young adult cancer survivors exposed to chest radiotherapy may have more pulmonary nodules than the general population, and pulmonary nodules are not limited to those survivors with a history of tobacco exposure. In addition, these findings suggest the possibility that as our cancer survivor cohort ages, the incidence of nodules will increase.

Given the heightened risk of lung cancer in radiation-exposed cancer survivors, lung cancer screening could theoretically be beneficial. Recently revised NCCN guidelines consider individuals with lymphoma at higher risk for lung cancer and suggest screening if age>50 and smoking history>20 PY [17]. Wattson et al used a Markov model approach to assess lung cancer screening in Hodgkin lymphoma survivors. They found screening with LDCT to be cost-effective only for smokers (defined as >10 PY) starting 6–20 years post radiation [18]. One study reported on low-dose chest CT for lung cancer screening in cancer survivors who were referred to lung cancer screening based on their smoking history (all had >30 PY) [19]. In this report, 30% had an abnormal scan. Less than half of the patients included in the study had received radiotherapy to the thorax or the neck and the screening CT was at an interval of 1.6–426.4 months post-cancer diagnosis. Therefore, the benefit or harm of CT-based lung cancer screening described in these studies may not be relevant to radiation-exposed cancer survivors without smoking history, such as those described herein.

The proportion of patients in our cohort with benign pulmonary nodules was high. On the patient level, 193/338 (57%) of survivors who had a chest CT had at least one pulmonary nodule detected; only 13/193 (6.7%) were ultimately diagnosed with a malignancy involving the lungs. On the nodule level, 448 unique nodules were detected of which 435 had follow-up data; 19/435 (4.3%) were malignant. This high proportion of benign findings warrants consideration of whether CT-based lung cancer screening in radiation-exposed survivors is appropriate. False positive findings, which are common in screening studies, are exacerbated by a high baseline prevalence of benign findings. Of note, the false-positive rate in the National Lung Screening trial was 23.4% [2][20]. The harm associated with such a high false-positive rate could outweigh the benefit associated with screening. Harms of screening include additional scans to follow-up on nodules, invasive procedures for evaluation of nodules and anxiety related to scans and screening.

Some other findings are noteworthy. The prevalence of a pulmonary nodule increased as time from diagnosis lengthened. This could be explained by a known increase in pulmonary nodule incidence with age or could correspond to late radiation-induced changes [14]. Not surprisingly, smoking history influenced risk of pulmonary nodules, especially substantial use (>30 PY). This is in concordance with previous findings on association between smoking and incidence of pulmonary nodules [21]. Survivors were more likely to have nodules on imaging if the decade of CT was more recent. This could be attributed to better resolution of CT scans, allowing for detection of more nodules, as has been shown previously [22][14]. In the multivariate analysis, splenectomy was also associated with a 57% increase in the risk of nodule. This has not been described before. One could speculate that splenectomy could increase the risk of infectious complications, resulting in inflammatory nodules in the lung.

We did not find an association of radiotherapy dose with risk for pulmonary nodules. It is possible that other factors such as radiation field or volume are more relevant to the severity of damage to the lungs than dose, or that we did not have the power to detect a difference by radiotherapy dose due to the narrow range of delivered doses, especially among older survivors. Another interesting finding is the high percentage of cancer survivors still undergoing chest CTs even more than 5 years after diagnosis. The timeframe of these scans suggest that they were not performed solely for recurrence monitoring and may have been indicated for pulmonary symptoms. In addition, most survivors had more than one chest CT with the range of CTs being 1–35.

Several limitations of our study need to be considered. In addition to its retrospective nature, this study describes survivors followed by a single institution’s high-risk survivorship clinic, which may limit the generalizability of the findings. In addition, we may be overestimating the rate of nodules if there was a selection bias for the survivors undergoing chest CTs. For example, survivors exhibiting pulmonary symptoms which are also in correlation with CT findings (such as infections). However, over half of our cohort had imaging performed, even in the case of some overestimation, the prevalence of pulmonary nodules would still be substantial. The strengths of this study are the extended time from diagnosis, allowing long-term outcomes to be assessed and detailed treatment exposure data.

In conclusion, this review of chest CTs among 338 survivors of childhood and young adult cancer with a history of radiotherapy affecting the lung demonstrated a high prevalence of benign pulmonary findings. In the setting of a CT-based lung cancer screening program, the high baseline prevalence of benign findings is likely to increase the risk of false positive findings and unnecessary procedures. Therefore, these results are critical to evaluating whether CT is appropriate for detection of lung cancer in survivors with a history of radiotherapy.

## Figures and Tables

**Table 1:** Demographic, diagnosis, and treatment information among adult survivors of childhood and young adult cancer with a history of radiation therapy to the chest

Demographics	All survivors (N=590)		Survivors with chest CT (N=338)	
Male (N,%)	278	47.1	153	45.3
**Median age at diagnosis (range)**	17.1 (0.4–39.8)		18.4 (0.4–39.8)	
**Median time since diagnosis, in years (range)**	21.1 (0.4–58.6)		23.3 (3.3–58.6)	
**Median attained age at last follow-up (range)** *	38.0 (17–80)		43 (17–80)	
**Smoker**				
Ever (N, %)	92	15.6	59	17.5
< 30 PY	68		46	
≥ 30 PY	6		4	
Unknown PY	19		9	
Never (N, %)	497	84.2	279	82.5
Unknown	1	0.2	0	0
**Diagnosis**				
Hodgkin Lymphoma (N, %)	291	49.3	210	62.1
Non-Hodgkin Lymphoma (N, %)	37	6.3	25	7.4
Sarcoma (N, %)	39	6.6	25	7.4
Leukemia (N, %)	99	16.8	35	10.4
Other (N, %)	124	21.0	43	12.7
**Treatment exposures**				
Hematopoietic cell transplant (N, %)				
None	404	68.5	246	72.8
Autologous (N, %)	51	8.6	34	10.1
Allogeneic (N, %)	135	22.9	58	17.1
**Radiation therapy field (median maximum dose, range)**				
Total body irradiation [13.7 Gy, 2–20] with or without other fields (N, %)	146	24.8	61	18.1
Mantle [36 Gy,6–50], with or without other fields, excluding TBI (N, %)	220	37.3	158	46.7
Whole Lung [15.0 Gy,12–55.8], with or without other fields, excluding TBI or mantle (N, %)	20	3.4	13	3.8
Mediastinal [30.6 Gy,5–60], with or without other field excluding all above (N, %)	101	17.1	70	20.7
Others [30.6 Gy, 51–59.4] (N, %) ***	103	17.5	36	10.5
**Chemotherapy**				
None (N, %)	64	10.9	36	10.6
Alkylating agents (N, %)	459	77.8	274	81.1
Unknown/missing (N, %)	67	11.3	28	8.3
**Splenectomy**				
Yes (N, %)	87	14.8	61	18.1
No/Unknown (N, %)	503	85.2	277	81.9

* Last follow-up was either last adult LTFU visit or last chest CT.

** To account for patients who had multiple chest radiation fields, we defined fields in a step-down manner. Each patient was included in only one row which corresponds to the primary chest radiation field. Each row allows exposure to any of the fields listed under it, but not above it. For example, patients counted in the row for the whole lung irradiation could have received mediastinal field irradiation or another field included in the others category but did not receive total body irradiation or mantle field.

*** Other fields include axilla, thoracic spine, chest wall, hemithorax, other lung.

Abbreviations: CT, computed tomography; TBI, total body irradiation; PY, person years

**Table 2:** Prevalence of selected abnormal chest CT findings among 338 adult survivors of childhood and young adult cancer with a history of radiation to the chest who had at least one chest CT

	Number of survivors	Percent (CI)
Any abnormality	271	80.1 (75.4–84.2)
Nodule	193	57.1 (51.6–62.4)
Opacity	127	37.5 (32.4–43.0)
Fibrosis	165	48.8 (43.3–54.3)

**Table 3:** Clinical outcomes following a pulmonary nodule among 193 adult survivors of childhood and young adult cancer with a history of radiation to the chest

	Number of survivors
**Procedure**	
Lung Biopsy	18
Benign pathology	10
Malignant pathology	8
**Diagnosis**	
Lung cancer diagnosed with lung biopsy	
Adenocarcinoma	4
Metastasis diagnosed with lung biopsy	
Metastasis of primary diagnosis (recurrence)*	2
Metastasis of secondary malignancy**	2
Metastasis diagnosed without lung biopsy	
Metastasis of secondary malignancy***	5
**No lung biopsy performed**	180

* Primary malignancies include: synovial sarcoma, rhabdomyosarcoma.

** Secondary malignancies include: Non-Hodgkin lymphoma, undifferentiated sarcoma.

*** Secondary malignancies include: head and neck squamous cell carcinoma, renal cell carcinoma, rectal carcinoma, cholangiocarcinoma, Ewing sarcoma.

**Table 4:** Risk factors associated with a pulmonary nodule on chest CT (827 chest CTs included)*

		Univariate			Multivariable**		
Demographics	% with 1^st^ pulmonary nodule	PR	95% CI	P	PR	95% CI	P
**Gender**				0.10			0.207
Male	20.1	0.77	0.56–1.05		0.81	0.59–1.12	
Female	25.9	Ref	--				
**Age at CT**				0.02			0.37
Per 10-year increase		1.16	1.02–1.32		1.07	0.92–1.24	
**Decade of CT**				<.01			0.03
1980–1990	11.1	0.33	0.05–2.40		0.35	0.04–2.92	
1991–2000	15.9	0.47	0.31–0.74		0.51	0.31–0.82	
2001–2010	21.9	0.66	0.49–0.89		0.69	0.51–0.94	
2011 +	33.2	Ref			Ref	Ref	
**Smoker**				0.08			
Ever	29.6	1.34	0.96–1.87				
Never	22.1	Ref	--				
**By pack year**				0.056			
<30 PY	28.4	1.28	0.89–1.85	0.17	1.18	0.79–1.75	0.40
30+ PY	80.0	3.61	2.47–5.29	<.01	3.39	1.92–6.00	<0.01
Never	22.1	Ref			Ref		
**Diagnosis**				0.49			
Hodgkin Lymphoma	23.3	Ref	--				
Non-Hodgkin Lymphoma	28.0	1.20	0.78–1.83				
Sarcoma	19.4	0.83	0.42–1.64				
Leukemia	30.6	1.31	0.82–2.08				
Other	19.3	0.84	0.55–1.28				
**Treatment exposures**							
**Hematopoietic cell transplant**				0.11			0.30
Allo	25.0	1.00	0.69–1.45		1.08	0.69–1.67	
Auto	14.0	0.56	0.33–0.95		0.68	0.38–1.20	
No	24.8	Ref	--				
**Radiation therapy field**				0.82			
TBI	25.3	1.17	0.78–1.73				
Mantle	21.7	Ref	--				
Mediastinal	24.1	1.11	0.77–1.59				
Whole Lung	32.0	1.47	0.75–2.90				
Other fields	24.3	1.12	0.65–1.91				
**Maximum Radiation dose**				0.84			
1–1000 cGy	15.5	Ref	--				
1001–2000 cGy	24.5	1.57	0.73–3.41				
2001–3000 cGy	23.2	1.49	0.70–3.14				
3001–4000 cGy	22.6	1.45	0.70–3.02				
>4000 cGy	21.0	1.35	0.58–3.12				
**Chemotherapy**				0.98			
Alkylating agents	23.1	0.99	0.64–1.56				
Without alkylating agents	23.1	Ref	--				
**Splenectomy**							0.016
Yes	31.2	1.43	1.04–1.96	0.02	1.57	1.09–2.25	
No	21.8	Ref	--				

* GEE methods with independent covariate matrix to account for cluster data.

** Variables with p<=0.2 were included in the multivariable model

Abbreviations: CT, computed tomography; PR, prevalence ratio; TBI, total body irradiation; PY, person years

## Abbreviations

CT: computed tomography
CI: confidence interval
LTFU: Long-Term Follow-Up
LDCT: low-dose computed-tomography
MSKCC: Memorial Sloan Kettering Cancer Center
PY: pack-years
PR: prevalence risk
USPSTF: United States Preventive Service Task Force
